# Gastric partitioning *versus* gastrojejunostomy for gastric outlet obstruction due to unresectable gastric cancer: randomized clinical trial

**DOI:** 10.1093/bjsopen/zrae152

**Published:** 2025-01-21

**Authors:** Marcus Fernando Kodama Pertille Ramos, Marina Alessandra Pereira, André Roncon Dias, Osmar Kenji Yagi, Bruno Zilberstein, Ulysses Ribeiro-Junior

**Affiliations:** Department of Gastroenterology, Hospital das Clinicas HCFMUSP, Faculdade de Medicina, Universidade de Sao Paulo, São Paulo, Brazil; Department of Gastroenterology, Hospital das Clinicas HCFMUSP, Faculdade de Medicina, Universidade de Sao Paulo, São Paulo, Brazil; Department of Gastroenterology, Hospital das Clinicas HCFMUSP, Faculdade de Medicina, Universidade de Sao Paulo, São Paulo, Brazil; Department of Gastroenterology, Hospital das Clinicas HCFMUSP, Faculdade de Medicina, Universidade de Sao Paulo, São Paulo, Brazil; Department of Gastroenterology, Hospital das Clinicas HCFMUSP, Faculdade de Medicina, Universidade de Sao Paulo, São Paulo, Brazil; Department of Gastroenterology, Hospital das Clinicas HCFMUSP, Faculdade de Medicina, Universidade de Sao Paulo, São Paulo, Brazil

## Abstract

**Background:**

Gastric outlet obstruction due to unresectable tumours is usually managed with a gastrojejunostomy. Unfortunately, the unsatisfactory outcomes of this procedure have led to the search for alternatives, including gastric partitioning.

**Methods:**

Monocentric, randomized, parallel, open-label trial that included patients with obstructive, unresectable distal gastric tumours. The objective was to compare gastric partitioning to gastrojejunostomy, considering the gastric outlet obstruction scoring system scale as the main outcome. Randomization was performed using computer-generated software available online and after the application of the informed consent term, the allocation group was revealed to the surgeon before the surgical procedure.

**Results:**

Over 7 years, 90 patients were initially randomized. After applying the inclusion and exclusion criteria, 25 patients were included in the gastrojejunostomy group and 27 in the partitioning group. Both groups were similar regarding initial clinical characteristics including sex, age, weight, clinical performance, and the acceptance of oral diet. Surgery duration, length of hospital stay, postoperative complications, and 30- and 90-day mortality rates were similar between groups. Acceptance of normal diet was more frequently reached by patients in the partitioning group (96% *versus* 72%; *P* = 0.022). During outpatient follow-up, maintenance of oral intake and weight was similar between groups. Patients in the partitioning group received more frequent red blood cell transfusions (81% *versus* 52%; *P* = 0.024). There was no difference regarding the administration of palliative chemotherapy lines and survival. In the multivariable analysis, the inability to eat a full diet (*P* = 0.035) and the absence of palliative chemotherapy after the procedure (*P* = 0.001) were associated with worse survival.

**Conclusions:**

Gastric partitioning provided a better return of the ability to accept food orally. There was no difference regarding postoperative complications and long-term survival.

**Trial registration:**

NCT02065803, clinicaltrials.gov

## Teaser

In this RCT that included 52 patients, patients who underwent gastric partitioning were more able to ingest a normal diet after the procedure (96.3% *versus* 72%, *P* = 0.022) with similar postoperative complications and survival. Gastric partitioning should be considered as the primary option for the treatment of malignant gastric outlet obstruction.

## Introduction

Even though stage IV gastric cancer (GC) is out of reach of curative treatment, many patients require surgical palliative treatment for complications related to tumour progression. These complications include bleeding, perforation, and distal gastric outlet obstruction (GOO). The incidence of GOO ranges between 5% and 14.9% in patients with distal GC. Whenever possible, in patients who present favourable clinical conditions, palliative resection should be performed.^1–4^ However, some of these tumours are considered unresectable due to local invasion or poor patient performance.^5,6^ In this setting, surgical bypass or endoscopic stents are options to restore gastroduodenal continuity. Endoscopic stents have the advantage of being less invasive, but as their long-term patency is inferior, their main indication is for patients with limited life expectancy.^7,8^

Surgical bypass is traditionally performed through a gastrojejunostomy, but about 10–26% of patients develop delayed gastric emptying (DGE), which prolongs the length of hospital stay and may affect the tolerance of palliative chemotherapy.^9–11^ Gastric partitioning associated with gastrojejunostomy has been considered a potential alternative possibly alleviating DGE. It was initially described for the treatment of complex gastroduodenal ulcers and its indication was further extended for unresectable tumors.^12,13^ It is currently already considered an option for palliative surgery by the Japanese Gastric Cancer Association.^14^

Previous retrospective studies demonstrated its benefit compared to gastrojejunostomy for return of oral intake, in addition to other advantages such as less need for red blood cell (RBC) transfusions, greater adherence to palliative chemotherapy, and improvement in survival. In order to provide high-level evidence regarding the benefits of gastric partitioning, we designed and conducted an randomized clinical trial (RCT) to compare gastrojejunostomy to gastric partitioning in addition to gastrojejunostomy for the treatment of GOO due to obstructive unresectable distal GC.^15^

## Methods

### Patients and study design

This was a prospective, randomized, parallel, open-label trial, carried out in a single centre. Patients aged between 18 and 85 years with unresectable gastric adenocarcinoma, and obstructive symptoms were considered eligible for the study. Further inclusion criteria were Eastern Cooperative Oncology Group (ECOG) 0-1-2, with an expected survival greater than 2 months. Patients were excluded if they had proximal GC or tumours involving the lesser curvature, proximal to the incisura angularis, tumours that invaded the greater curvature above the middle third of the stomach, obstructive condition originating in the small bowel or colon, and peritoneal carcinomatosis index greater than 12 evaluated during the surgical procedure.

Obstructive symptoms were graded according to the Gastric Outlet Obstruction Score (GOOS) as follows: 0 = no oral intake, 1 = liquid only, 2 = soft solids, 3 = low residue or full diet.^16^ Patients with GOOS ≤ 2 associated with early bloating and vomiting were considered as obstructed.

Pretreatment clinical staging was performed by abdominal, and pelvis computed tomography (CT) and upper digestive endoscopy. Baseline clinical characteristics included sex, age, weight, body mass index (BMI), ECOG performance status, Karnofsky performance scale, American Society of Anesthesiologists (ASA) score, Charlson–Deyo Comorbidity index (CCI), and laboratory tests.

### Randomization and masking

The randomization was carried out by the hospital’s research centre using computer-generated software available online (www.random.org). A spreadsheet with a sequence of 100 numbers with the hidden letter of the corresponding group was generated and maintained in the hospital data centre without access to study participants. The inclusion of patients was performed during outpatient appointments or hospitalization by the surgeon responsible for conducting the case. After application of the informed consent term, the allocation group was revealed to the surgeon.

### Procedures

**Fig. 1:**
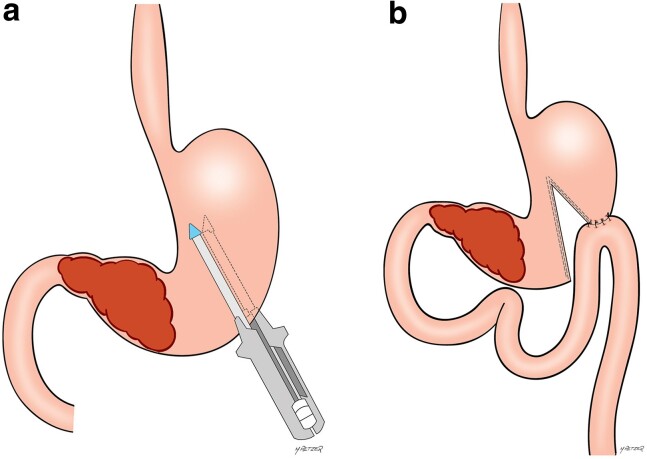
Gastric partitioning surgical scheme **a** Positioning of the stapler proximal to the gastric tumour. **b** Final aspect after the partial gastric transection with the gastrojejunostomy on the proximal gastric chamber.

The access route could be open or laparoscopic, according to the surgeon’s preference. Confirmation of the impossibility of resection was performed during the surgical procedure. If the possibility of removing the tumour was verified during the procedure, it was performed and the patient was excluded. The gastric partitioning technique employed was previously described.^17^ Briefly, upon confirmation that the tumour was unresectable, the lesser sac was accessed, and the posterior gastric wall was inspected to confirm that there was a tumour-free area for the anastomosis. A point located at least 5 cm proximal to the tumour along the gastric curvature was chosen. A 32 Fr bougie was positioned along the lesser curvature to ensure a small conduit between the two gastric chambers created by the partitioning. The stomach was then divided through a mechanical linear stapler from the greater curvature towards the bougie along the lesser curvature. A side-to-side gastrojejunostomy, 30 cm from the ligament of Treitz, was performed along the greater curvature of the proximal gastric chamber (*Fig. 1*). After both procedures, the oral nasogastric tube was removed when the daily output was less than 500 ml in 24 h and the patient had bowel sounds. Liquid diet was subsequently introduced, and further progression occurred according to the patient’s acceptance.

### Outcomes

The primary endpoint of the study was the patient-centred acceptance of oral intake according to the GOOS scale after the procedure, with the GOOS 3 value at discharge being considered technical success of the procedure. Secondary endpoints were duration of surgery, surgical complications (graded according to the Clavien–Dindo classification)^18^, 30- and 90-day mortality, red blood cell (RBC) transfusions, adherence to palliative chemotherapy, and overall survival.

All patients were evaluated bimonthly following surgery. In all appointments, weight, oral diet acceptance (GOOS), and transfusion of RBC were checked. Palliative chemotherapy was prescribed by the clinical oncology staff. The absence of appointments for more than 6 months was considered a loss to follow-up.

### Sample size

The sample size calculation assumed that the proportion of patients with an inability to eat food after the procedure (GOOS ≤ 2) would be 25% with gastrojejunostomy (GJ) and 0% with gastric partitioning in addition to gastrojejunostomy (GP). Therefore, to provide 80% power to detect this improvement in achieving a GOOS 3, a sample size of 52 patients was required (significance level of *P* < 0.05).

As the patients involved in the study usually undergo frequent follow-ups in the hospital, a follow-up loss of less than 10% was expected. On average, our institution performed 15 gastrojejunostomy procedures per year.^15^ Considering that not all patients would meet the inclusion criteria and that the expected median survival of this population would be 180 days, it was expected to complete the recruitment in 5 years and the study in 6 years. No interim analysis was planned.

### Statistical analysis

Parametricity was determined using the Shapiro–Wilk test, with normally distributed data being expressed as mean(s.d.) and non-parametric data as median (i.q.r.). Comparison between quantitative variables was performed using the Student’s *t*-test or the Mann–Whitney U test, and chi-square or Fisher’s exact test was used for categorical variables. The association of the surgical procedure with the occurrence of primary and secondary outcomes was analysed by binary logistic regression analysis, and odds ratios with 95% c.i. were calculated.

Survival analysis was performed using the Kaplan–Meier method, and the difference between the curves was examined by the log-rank test. Factors associated with overall survival (OS) were estimated using the Cox proportional hazards model, and hazard ratios with a 95% confidence interval were calculated to determine which variables were independently related to prognosis. Variables with *P* < 0.250 were included in the multivariable model. All analyses were performed using SPSS version 20.0 statistical program (SPSS, Chicago, IL, USA). *P* < 0.05 were considered statistically significant.

### Ethical issues

The present study was approved by the Institution’s Research Ethics Committee and registered on the national research platform and clinicaltrials.gov (NCT02064803).

## Results

**Fig. 2:**
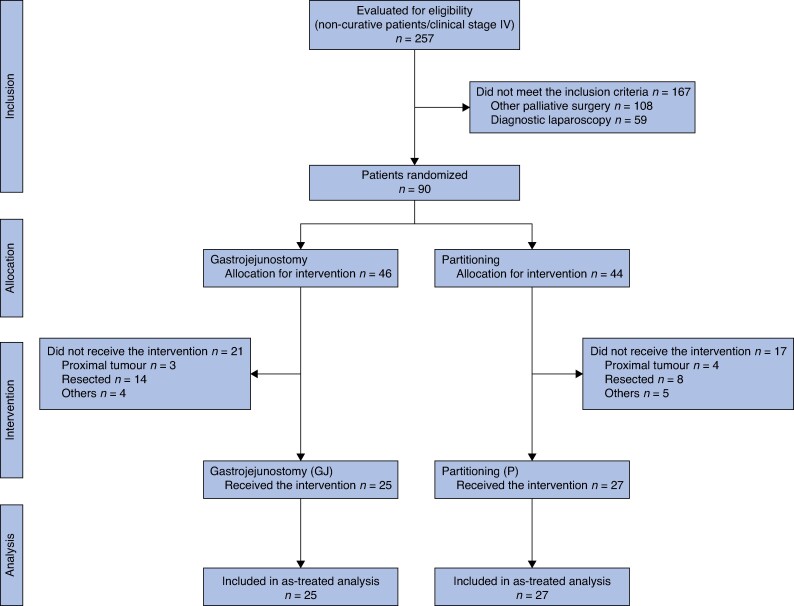
Flowchart of the study

Between 1 August 2013 and 15 April 2020, 816 patients with GC were referred for surgical treatment at our institution. Among these, 90 of the 257 patients with non-curative stage IV GC assessed for eligibility were enrolled in the study: 46 patients were randomized to gastrojejunostomy (GJ group) and 44 to gastric partitioning in addition to gastrojejunostomy (GP group). After the exclusions, 25 patients in the GJ group and 27 patients in the GP group were included in the final analysis. The study flowchart is shown in *Fig. 2*.

The baseline characteristics of the 52 patients included in the final analysis are shown in *Table 1*. There was no significant difference between the groups for the evaluated characteristics, including sex, age, BMI, initial GOOS classification, and clinical performance status. The most frequently invaded adjacent structure, which prevented surgical removal of the tumour, was the pancreas and duodenum, occurring in 80% and 89% of the cases in the GJ group and the GP group respectively. The presence of metastases was observed in 64% of the cases in the GJ group and 56% of the cases in the GP group.

**Table 1:** Baseline and tumour characteristics

Variables	Gastrojejunostomy	Partitioning
	*n* = 25	*n* = 27
**Sex**		
Female	4 (16)	10 (37)
Male	21 (84)	17 (63)
Age (years), mean(s.d.)	61.4(11.7)	66.1(8.1)
BMI (kg/m^2^), mean(s.d.)	20.7(4.0)	21.3(3.4)
**ECOG scale**		
1	18 (72)	15 (55.6)
2	7 (28)	12 (44.4)
**Karnofsky performance scale**		
60–70	7 (28)	9 (33.3)
80–90	18 (72)	18 (66.7)
**Laboratory, mean(s.d.)**		
Haemoglobin (g/dl)	10.1(1.4)	9.7(1.7)
Albumin (g/dl)	3.5(0.5)	3.5(0.6)
Neutrophil-to-lymphocyte	3.25(1.5)	4.17(3.5)
**Initial GOOS**		
0	7 (28)	6 (22.2)
1	15 (60)	18 (66.7)
2	3 (12)	3 (11.1)
**Metastasis**		
Peritoneal	13 (52)	10 (37)
Hepatic	8 (32)	5 (18.5)
M1	16 (64)	15 (55.6)

Values are *n* (%) unless otherwise indicated. BMI, body mass index; ECOG, Eastern Cooperative Oncology Group; GOOS, Gastric Outlet Obstruction Score.

There was no difference between the groups regarding the duration of the procedure, postoperative complications, and mortality rate within 30 and 90 days (*Table 2*). Only three cases underwent laparoscopic surgery and all cases had gastrojejunostomy without Roux-en-Y. Regarding the GOOS score after surgery, we found that GOOS 3 was reached following surgery in 26 patients (96%) of the cases in the GP group, compared to 18 patients (72%) of the cases in the GJ group (*Table 3*). Only two patients had final values of GOOS 0 and GOOS 1 (1 in GJ and 1 in GP). There was no difference between the groups regarding the time required to reach GOOS 1 and 2 values. The mean duration of acceptance of a GOOS ≥ 2 diet was 220.3 days for the GJ group and 320.6 days for the GP group (*P* = 0.68).

**Table 2:** Surgical, postoperative outcomes, and oral intake after the procedure

Variables	Gastrojejunostomy	Partitioning	*P*
	*n* = 25	*n* = 27	
Length of hospital stay (days) meadian (i.q.r.)	5 (4–5.5)	6 (4–8)	0.38
**Final GOOS**			**0.005**
0	1 (4.2)	0 (0)	
1	0 (0)	1 (3.7)	
2	6 (25)	0 (0)	
3	18 (72)	26 (96.3)	
Days to reach GOOS 2*, mean(s.d.)	3.5(1.6)	3.9(1.5)	0.395
Duration of oral intake GOOS ≥2 (days)*, mean(s.d.)	220.3(406.6)	320.6(238.7)	0.680
BMI (kg/m²)—maximum, mean(s.d.)	21.9(3.8)	23.3(3.5)	0.175
**Status—weight 30 days**			0.438
Weight loss	12 (66.7)	13 (54.2)	
Weight gain	6 (33.3)	8 (33.3)	
Weight maintenance	0 (0)	3 (12.5)	
**Status—weight 90 days**			0.242
Weight loss	10 (66.7)	8 (36.4)	
Weight gain	4 (26.7)	10 (45.5)	
Weight maintenance	1 (6.7)	4 (18.2)	
**Palliative chemotherapy**			
1st line	15 (60)	22 (81.5)	0.088
2nd line	6 (24)	10 (37)	0.309
3th line	4 (16)	2 (7.4)	0.411

Values are *n* (%) unless otherwise indicated. *Only patients who reached this value were included. Bold numbers represent *P* < 0.005. GOOS, Gastric Outlet Obstruction Score; BMI, body mass index.

**Table 3:** Assessment of the primary and secondary outcomes of the study, in relation to the surgical arm—partitioning *versus* gastrojejunostomy

Outcomes	Gastrojejunostomy	Partitioning	OR	95% c.i.	*P*
	*n* = 25	*n* = 27			
GOOS 3 at discharge	18 (72)	26 (96.3)	10.11	1.14,89.43	**0.037**
Duration of surgery (min), mean(s.d.)	61.3(19.0)	75(40.1)	1.01	0.99,1.04	0.145
Major surgical complications	2 (8)	2 (7.4)	0.92	0.12,7.08	0.936
30-day mortality	3 (12)	1 (3.7)	0.28	0.03,2.91	0.288
90-day mortality	8 (32)	5 (18.5)	0.48	0.13,1.74	0.267
RBC transfusions	13 (52)	22 (81.5)	4.06	1.17,14.15	**0.024**
Adherence to palliative chemotherapy—1st line	15 (60)	22 (81.5)	2.93	0.83,10.32	0.094
Overall survival (median)	5.3	12.4	1.43*	0.80,2.56	0.154

Values are *n* (%) unless otherwise indicated. * Hazard ratio. Bold numbers represent *P* < 0.005. GOOS, Gastric Outlet Obstruction Score; RBC, red blood cells.

The evolution of the patient’s weight after the procedure showed that more than half of the patients had lost weight after 30 days compared to the initial value in both groups. After 90 days, 46% of patients in the GP group had gained weight, whereas 67% of patients in the GJ group had lost weight (*P* = 0.242). During outpatient follow-up, in both groups, most patients had the maximum weight measured above the initial value. On the other hand, the last measured weight of patients before death was lower than the initial weight in most cases, also with no difference between groups.

During outpatient follow-up, the rate of patients who received RBC transfusion was higher in the GP group (81.5% *versus* 52%; *P* = 0.024). The rate of RBC transfusion per month of follow-up did not differ between groups (*P* = 0.727). There was no difference between groups regarding the palliative chemotherapy lines given to patients. The type of chemotherapy did not differ between the groups, with the most frequently administered regimen being FLOX (fluorouracil–leucovorin–oxaliplatin).

**Fig. 3:**
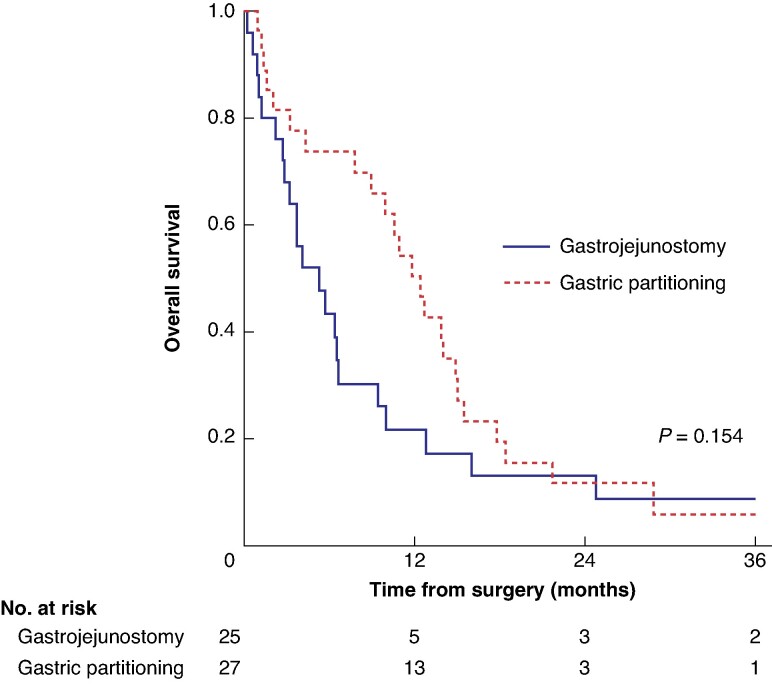
Overall survival stratified by gastric partitioning *versus* gastrojejunostomy only groups

The median OS for the entire cohort was 8.9 months. During the follow-up period, 49 patients died. The median survival was 5.3 months for the GJ group and 12.4 months for the GP group (*P* = 0.154; *Fig. 3*).

Univariable and multivariable analyses of factors associated with OS are presented in *Table 4*. Failure to undergo chemotherapy after the procedure (*P* = 0.001) and a final GOOS < 3 (*P* = 0.035) were independent factors associated with worse survival.

**Table 4:** Univariable and multivariable analysis for overall survival

	Univariable			Multivariable*		
Variables	HR	95% c.i.	*P*	HR	95% c.i.	*P*
Age ≥65 years (*versus* < 65)	0.88	0.49,1.57	0.661	—	—	—
Male (*versus* female)	0.8	0.42,1.54	0.512	—	—	—
BMI <18.5 (*versus* ≥18.5)	0.89	0.45,1.76	0.743	—	—	—
ASA III (*versus* II)	1.49	0.84,2.66	0.173	0.75	0.36,1.56	0.435
Charlson Index ≥1 (*versus* 0)	0.76	0.47,1.75	0.903	—	—	—
ECOG 2 (*versus* 1)	1.18	0.65,2.12	0.589	—	—	—
Karnofsky 60–70% (*versus* 80–90%)	1.74	0.92,3.30	0.088	0.97	0.43,2.17	0.934
Haemoglobin <11 g/dl (*versus* ≥11)	1.62	0.77,3.40	0.203	1.46	0.63,3.38	0.382
Initial GOOS 0 (*versus* 1–2)	1.62	0.83,3.17	0.158	1.11	0.53,2.34	0.790
Peritoneal metastasis P1 (*versus* P0)	1.2	0.67,2.17	0.536	—	—	—
Final GOOS 0–1–2 (*versus* 3)	7.88	3.25,19.08	**<0.001**	3.58	1.10,11.72	**0.035**
Gastrojejunostomy (*versus* partitioning)	1.43	0.80,2.56	0.231	0.87	0.41,1.84	0.720
None (*versus* chemotherapy)	5.83	2.84,11.98	**<0.001**	4.97	1.85,13.32	**0.001**

*Variables with *P* < 0.250 were included in the multivariable model. Bold numbers represent *P* < 0.005. BMI, body mass index; ASA, American Society of Anesthesiologists; ECOG, Eastern Cooperative Oncology Group; GOOS, Gastric Outlet Obstruction Score.

## Discussion

The present study evaluated the clinical and surgical outcomes of patients who underwent gastrojejunostomy compared to gastric partitioning in addition to gastrojejunostomy for malignant GOO. The initial characteristics of both groups were similar, including the acceptance of oral diet. The primary endpoint of the study was the assessment of oral diet acceptance using the GOOS scale after the procedure, which was significantly higher for patients who underwent gastric partitioning.

The GOOS score reached by the patient after the procedure was chosen as the primary endpoint of the study. The GOOS scale was initially described by Adler and Baron to evaluate the results of the use of metallic prostheses for the treatment of GOO.^16^ Due to its simplicity, easy reproduction, and correlation with relevant clinical results, it started to be widely used.^9,19^ Another advantage of choosing the GOOS value after the procedure as the primary endpoint is that its effect would be evident in the short term during hospitalization. This prevents any loss of follow-up from limiting the results obtained. On the other hand, the long-term effectiveness of the procedure is not evaluated. For this reason, to complement the analyses, we included late secondary outcomes, such as adherence to the chemotherapy, and maintenance of the GOOS value and OS.

For the adoption of a new surgical procedure, safety assessment is fundamental. One concern of the technique was whether the partitioning could add to the risk of stomach staple line dehiscence. Another concern was the possible failure of retrograde emptying from the distal gastric chamber to the proximal one, which could lead to rupture of the distal gastric part. In the present study, the groups did not differ in terms of the occurrence of postoperative complications in agreement with previous reports.^9,20,21^ Additionally, there was no difference between the techniques regarding the duration of surgery. At first, the duration of both procedures may seem high for a simple procedure. However, it is important to emphasize that the attempt to resect the tumour was allowed before proceeding with the gastric bypass, which was also the main reason for excluding the cases initially randomized for the study.

As previously mentioned, patients with poor performance and a life expectancy of less than 2 months may benefit from treatment using endoscopic prostheses.^6,22^ In the present study, patients undergoing partitioning had a 30-day mortality rate of 3.7% and a 90-day mortality rate of 18.5%, demonstrating a good selection of patients for participation in the study. One of the study exclusion criteria was low performance defined as ECOG 3 and 4, precisely to avoid performing bypass surgery in these patients.

In addition to food intake, the assessment of the clinical status of patients during follow-up also included the monitoring of weight.^23^ Weight evolution after the procedure showed an initial drop in the first 30 days in both groups. As for long-term weight outcomes, patients in both groups reached a higher weight at some point, defined in the study as maximum weight. However, as the disease progressed, there was also a progressive weight loss. Consequently, the final weight of each patient was in most cases lower than the initial weight measured before the procedure. Despite this progressive weight loss, the maintenance of acceptance of the oral diet was prolonged in both groups, showing the duration of the benefits of the palliative surgery, regardless of the technique performed.^24^

One of the hypotheses of the study was that, when performing the partitioning and isolating the tumour in the distal gastric chamber, the tumour would not have contact with the ingested food, thus reducing the occurrence of tumour bleeding and the need for transfusion of RBC.^24^ An overall analysis yielded that the gastrojejunostomy group received fewer transfusions than the partitioning group. However, when analysing the number of transfusions according to the number of months of follow-up for each patient, there was no difference between groups. This was an exploratory analysis that was not initially planned, but which we thought was pertinent to be carried out.

Regardless of the technique employed, bypass surgery also aims to improve the patient’s clinical conditions so they are more prone to receive palliative chemotherapy. In the present study, there was no difference between the rate of patients who received first-, second-, and third-line chemotherapy between the groups. The analysis of factors related to overall survival confirmed the importance of palliative chemotherapy as an independent factor associated with better survival, together with adequate oral food intake. It is noteworthy that more than 70% of patients included received first-line chemotherapy, once again reflecting the adequate selection of cases for inclusion in the study.

OS was assessed as a secondary endpoint. As the sample calculation was performed for GOOS assessment (planned main outcome), the number of patients included may be insufficient for comparing long-term survival between the two techniques. Although the group submitted to gastric partitioning had a better median survival compared to the gastrojejunostomy group, the difference was not significant. This result was similar to that presented in the meta-analysis by Lorusso *et al*., where a trend towards better survival results was verified in the partitioning group.^25^

The study has some limitations and biases that should be mentioned. First, a considerable number of patients were excluded after randomization. In the present trial, 38 patients were excluded, which corresponds to more than 50% of the cases included in the final analysis. Despite this high number of exclusions, it was found that more than half of the exclusions occurred due to tumour resection. As many times the definition of the impossibility of tumour resection could not be adequately defined preoperatively, all patients who met the other inclusion criteria were recruited and, if the tumour was resected, the patient was excluded.

In a randomized trial, by virtue of randomization, any difference in baseline characteristics will be due to chance; as there were many exclusions, this precept could have been broken. Fortunately, even with the exclusions, the initial characteristics in both groups were similar, justifying the final per-protocol analysis. Unfortunately, a blinded outcome assessment was not possible. However, multidisciplinary teams not directly linked to the study also participated in the assessment of late outcomes, minimizing the risk of bias.

To the best of our knowledge, this is the first RCT comparing the outcomes of gastrojejunostomy and partitioning for the treatment of GOO. Although the study was carried out in a department with great expertise, the simplicity of the technique ensures good external validity.

In conclusion, patients with obstructive distal gastric tumours who underwent gastric partitioning had better oral diet acceptance after the procedure. Both techniques had similar surgical and postoperative outcomes, with no significant difference in survival. A final GOOS 3 status and receiving some line of chemotherapy were independent factors associated with improved OS.

## Supplementary Material

zrae152_Supplementary_Data

## Data Availability

The data that support the findings of this study are not openly available and are available from the corresponding author upon reasonable request.
