# Supplementary material for: Gastric partitioning versus gastrojejunostomy for gastric outlet obstruction due to unresectable gastric cancer: randomized clinical trial
Source: BJS Open. 2025 Jan 21;9(1):zrae152. doi: 10.1093/bjsopen/zrae152 (PMC11747725; doi:10.1093/bjsopen/zrae152)

University Of São Paulo Medical School

Department of Gastroenterology

Randomized clinical trial comparing gastrojejunostomy with or without performing gastric partitioning for palliation of obstructive and unresectable gastric tumors.

Dr. Marcus Fernando Kodama Pertille Ramos

Dr. Osmar Kenji Yagi

Dr. André Roncon Dias

Prof. Dr. Ulysses Ribeiro Júnior

2014

1. Introduction

Cancer accounts for an increasingly larger portion of the causes of morbidity and mortality in the Brazilian population and this situation is expected to worsen in the coming decades with an increase in the incidence of the disease resulting from the aging of the population. The incidence (adjusted by age) of gastric cancer in the world is in fourth place, in both sexes. In terms of mortality (adjusted by age), it is second among males and third among females (Parkin et al., 2005). More than 70% of cases occur in developing countries. Furthermore, the incidence rate is about twice as high in males than in females. In 2012, there were an estimated 12,670 new cases of stomach cancer in men and 7,420 in women in Brazil. These values correspond to an estimated risk of 13 new cases for every 100,000 men and 7 for every 100,000 women. Without considering non-melanoma skin tumors, stomach cancer in men is the second most common in the North (11/100,000) and Northeast (9/100,000) regions and the fourth in the South (16/100,000) regions. Southeast (15/100 thousand) and Central-West (14/100 thousand). For women, it ranks fourth in the North region (6/100 thousand), fifth in the Central-West region (7/100 thousand), sixth in the Southeast (9/100 thousand), South (8/100 thousand), and Northeast (6/100 thousand).

The main non-curative therapeutic modality for the treatment of obstructive gastric cancer is surgery. Whenever possible, tumor resection without associated lymphadenectomy should be performed. Surgical resection presents greater durability of acceptance of the oral diet with improved quality of life. However, many patients have locally advanced tumors that cannot be resected. The incidence of patients with gastric cancer in this situation varies in the literature from 5 to 30%. In these cases, gastrointestinal bypass procedures can improve quality of life by relieving symptoms of difficulty in oral intake without presenting a high surgical risk. To quantify the intensity of gastric obstruction, Adler et al proposed in 2002 a classification called GOOSS – Gastric Outlet Obstruction Scoring System – with the following degrees:

0 = no oral intake

1 = liquid diet

2 = soft diet

3 = low residue or general diet

The traditionally most used procedure is gastro-entero anastomosis, also called gastrojejunostomy. This anastomosis is performed on the posterior wall of the stomach with the first jejunal loop that reaches the stomach without tension in a side-to-side manner with a wide extension. The anastomosis can be performed manually or mechanically. The procedure is simple and quick to perform. Its drawbacks are a frequency reported in the literature of 10 to 26% of cases in which there is difficulty in gastric emptying through the anastomosis. Another inconvenience arises from the maintenance of the tumor in contact with the ingested diet, predisposing it to a greater risk of tumor bleeding. Finally, there is a risk of new obstruction due to the growth of the tumor that is close to the anastomosis.

Exclusive complete gastric partitioning associated with a gastrojejunostomy was originally described by Devine in 1925 as a method of antral exclusion and complete division of the stomach accompanied by a gastrojejunostomy in the proximal gastric chamber for the management of difficult duodenal ulcers. Subsequently, Maingot et al used the same technique for the management of obstructive distal gastric tumors, calling it the Devine procedure. Due to the risk of rupture of the distal gastric stump, Kaminishi et al in 1997 described a modification of the surgery, maintaining a patent gastric segment of approximately 3 cm in the lesser curvature communicating the two gastric chambers that were separated by horizontal stapling of the stomach. This communication allows the reflux of gastric contents from the distal to the proximal chamber where the anastomosis with the small intestine is performed. In this way, the risk of rupture of the distal gastric chamber is avoided, the food ingested is in less contact with the tumor and the tumor can also be evaluated by endoscopy. This procedure is referred to in the literature as modified Devine exclusion, gastric partitioning, or gastric bipartition (figure 1). In this work, we will use the name gastric partitioning.

The technique can be performed by laparoscopy as reported in 2 cases operated by Ammori et al. in 2002. Laparoscopic partitioning was also later used by Matsumoto et al. in an obstructive duodenal tumor in 2005. Finally, Suzuki et al. reaffirmed the possibility of performing the laparoscopic partitioning, reporting in 2006 a series of 8 cases operated without complications with the maintenance of oral intake until the clinical outcome. Unlike other authors, Suzuki performs a “Roux-en-Y” gastrojejunostomy with the aim of avoiding symptoms caused by bile reflux into the proximal gastric chamber.

Figure 1 – Schematic drawing of gastric partitioning.


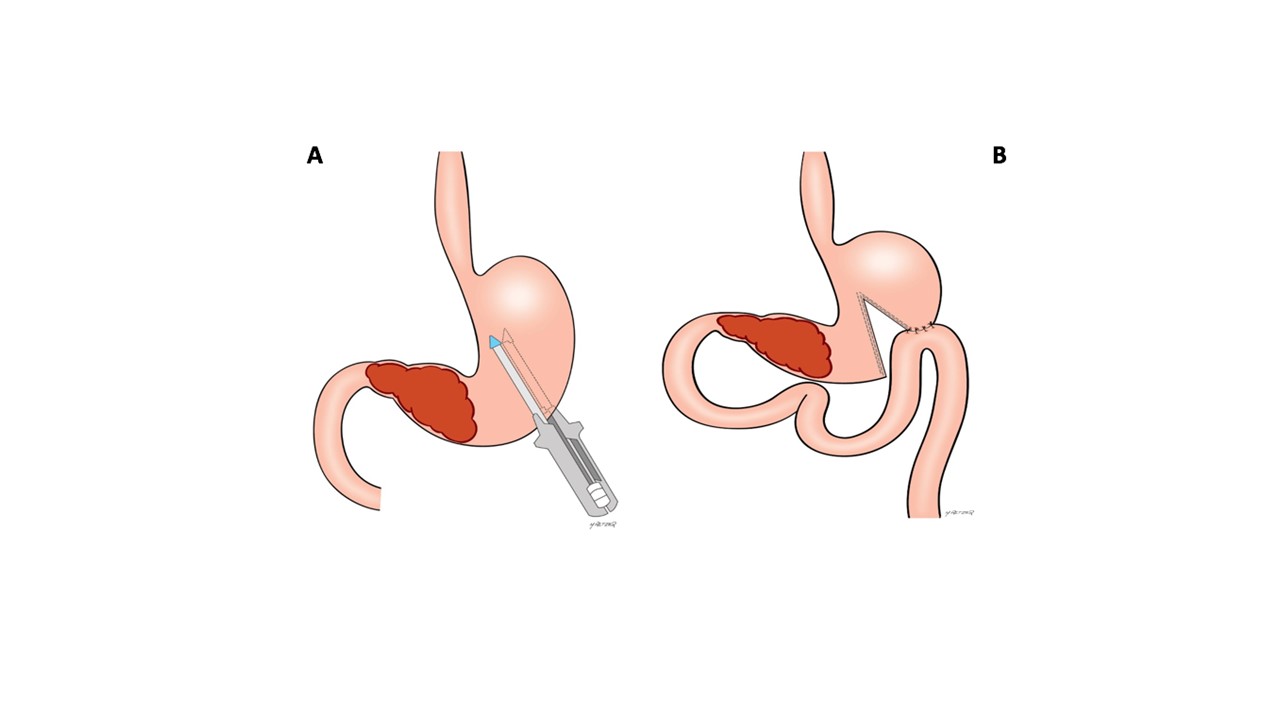


When comparing partitioning with gastrojejunostomy, Kwon et al. in 2004 found less weight loss, lower volume of blood transfusions, and better survival in a group of 18 patients undergoing partitioning compared with an equivalent group of patients undergoing gastrojejunostomy. Oida et al. published in 2009 a retrospective analysis comparing 30 patients undergoing gastrojejunostomy with 30 patients undergoing partitioning. Five cases of partitioning were performed laparoscopically. The technique used by Oida presented a variation as the partitioning was performed 3 cm close to the tumor followed by a new triangular resection of the proximal gastric chamber, enabling the performance of a horizontal side-to-side gastrojejunostomy. A Braun anastomosis 20 cm from the gastrojejunostomy was also performed. The results in favor of partitioning were significant, reaching statistical significance in the time required to remove the nasogastric tube (NGT), start of the diet, start of the solid diet, duration of hospitalization, stay at home without further hospitalization, and survival time. Recurrence of gastric obstruction did not occur in any case in the partitioning group compared to 5 cases (16.7%) of new obstruction that occurred in the gastrojejunostomy anastomosis group.

Gastric partitioning can also be used to treat obstructive peri-ampullary tumors. Arciero et al. applied the associated technique including bilio-digestive anastomosis in 20 cases with success. Usuba et al. used the technique in 46 patients, obtaining slightly lower morbidity than conventional gastrojejunostomy. Other authors such as Faroq and Yamagishi also used the technique for peri-ampullary tumors, demonstrating its applicability also for these types of tumors.

The use of endoscopic prostheses has also been frequently used for the palliation of gastric obstruction. In a recent study, carried out in our service and published by Moura et al., an improvement in GOOS was found in 15 patients with gastric and pancreatic neoplasia. However, the prosthesis has worse long-term results, as found by Jeurnink et al. after carrying out a multicenter randomized study (Sustent Study). The authors recommend the prosthesis for patients with low clinical performance with an Eastern Cooperative Oncology Group (ECOG) classification of 3 and 4 and a life expectancy of less than 2 months.

2-Objectives

Primary

- Evaluate whether gastric partition is more effective than gastrojejunostomy in improving and maintaining the patient's oral intake.

Secondary

- To evaluate whether patients undergoing partition have superior survival.

3-Materials and Methods

3.1 Study population

Patient with gastric adenocarcinoma who presents gastroduodenal obstruction and are not a candidate for surgical resection. Patients of both sexes aged between 18 and 85 years old will be considered eligible.

3.2 Participants

Dr. Marcus Fernando Kodama Pertille Ramos

http://buscatextual.cnpq.br/buscatextual/visualizacv.do?id=K4771312E1

Dr. Osmar Kenji Yagi

http://buscatextual.cnpq.br/buscatextual/visualizacv.do?id=K4771241Y6

Dr. Andre Roncon Dias

http://buscatextual.cnpq.br/buscatextual/visualizacv.do?id=K4496313P7

Prof. Dr. Ulysses Ribeiro Júnior

http://buscatextual.cnpq.br/buscatextual/visualizacv.do?id=K4708374T2

3.2 Study Design

This is a prospective randomized clinical trial in which patients will be randomly distributed into 2 groups. The first group (group A) will be considered the control group in which patients will undergo gastrojejunostomy. The second group (group B) will be considered the intervention group in which patients will undergo gastric partitioning. Randomization will be performed before the start of the study. Appropriate software will be used to generate a sequence of numbers with the letter of the corresponding group. The sequence of numbers will be maintained in the Digestive System Surgery service at the Cancer Institute of the University of São Paulo Medical School. After the patient is included in the study, the technique to be used, determined by the sequence, will be communicated to the surgeon responsible for the surgical procedure.

3.3 Ethical considerations.

The study project was submitted to the ICESP-FMUSP Ethics Committee (Cappesq) and registered online. Only individuals who signed the Free and Informed Consent Form (TCLE) will be included. The authors deny the existence of any conflict of interest.

3.4 Statistical Analysis

Statistical analysis will be performed using the Chi-square and Fischer tests for categorical variables and the non-parametric Mann-Whitney test for continuous variables. Kaplan-Meier survival curves will be used to define the difference in survival and maintenance of oral intake (endpoint) between the groups. For statistical analysis, we expect a reduction in the expected frequency of emptying delay of 26% for the absence of the event. The significance level will be considered 5%. The initial sample size calculation is 26 patients in each group. Considering that patients have an average survival of 120 days, we expect an improvement of more than 20% in the partition group. As the patients involved in the study usually undergo frequent follow-ups in the hospital, we expect a loss to follow-up of less than 10%. Statistical analysis will be performed using the SPSS program.

3.5 Inclusion criteria

- Patients with distal gastric tumors with obstructive conditions without indication for curative or palliative resection.

An obstructive condition is defined as GOOS equal to or less than 2 associated with early saciety and vomiting if the patient tries to maintain the usual volume of food intake.

- Confirmation that the obstructive condition is of gastroduodenal origin with imaging examination or Upper Digestive Endoscopy

- Absence of other points of obstruction distal to the lesion

- Histological diagnosis of gastric adenocarcinoma confirmed with biopsy

- Signing the informed consent form

3.6 Exclusion criteria

- Refusal to sign the consent form

- Tumors with an indication for curative or palliative resection

- Proximal gastric tumors or involving the lesser curvature proximal to the incisura angularis

- Tumors that invade the greater curvature above the middle third

- Patients with poor clinical performance with expected survival of less than 2 months – ECOG 3 and 4.

- Obstructive conditions originating in the small intestine or colon

- Diffuse peritoneal carcinomatosis with a peritoneal carcinomatosis index greater than 12.

- 3.7 Intervention

Patients underwent routine staging tests for gastric cancer consisting of:

- Upper Digestive Endoscopy

- Computed tomography scans of the chest, abdomen, and pelvis; Magnetic resonance imaging can be performed

- General laboratory tests

- Preoperative clinical assessment

The surgical procedure will only be performed by surgeons participating in the project with extensive experience in the surgical treatment of cancer of the gastrointestinal tract.

The gastrojejunostomy will be performed in a pre-colic, antiperistaltic manner on the posterior wall of the stomach with at least 5 cm of extension using the first jejunal loop approximately 30-40 cm from the angle of Treitz. The anastomosis may be manual or mechanical.

The gastric partitioning will be performed 3 to 5 cm proximally to the lesion in the greater curvature using a cutting linear stapler. Stapling will be carried out horizontally, preserving a narrow tunnel calibrated with a Bougie along the lesser gastric curvature. Subsequently, a gastrojejunostomy will be performed in a pre-colic, antiperistaltic manner on the posterior wall of the stomach with at least 5 cm of extension using the first jejunal loop approximately 40 cm from the angle of Treitz. The anastomosis may be manual or mechanical. Braun anastomosis will not be performed.

Patients in both groups will be maintained with a nasogastric tube (NGT). NGT will be removed when the daily output is less than 300 ml and a liquid diet will be introduced. The patient will be discharged after accepting a soft diet (GOSS=2). Delayed gastric emptying will be defined as the impossibility of removing the NGT after 3 days, the need to reintroduce the NGT, or the absence of swallowing soft foods 7 days after the surgical procedure. In cases with delayed gastric emptying, a contrast examination of the stomach will be performed.

4. Data collection

All data will be stored in a database based on the Access for Windows program. Demographic data, laboratory tests, staging tests, and surgical techniques will be collected before the procedure. Duration of surgery, blood loss, time to remove NGT, time to introduce liquid and soft diet, and length of stay will be evaluated at hospital discharge. Surgical complications will be reported using the Clavien-Dindo classification for surgical complications. During outpatient follow-up, data such as weight, length of stay at home, oral diet tolerance, bleeding episodes, and readmissions will be assessed at each appointment.

5. Follow-up

Patients will undergo outpatient follow-up every 2 months until death. No imaging tests or endoscopu will be routinely ordered. Patients will be advised to seek the hospital emergency service whenever they experience any complications.

6. Contact and Location

Dr. Marcus F. Kodama P. Ramos

email: marcus.ramos@icesp.org.br

Cancer Institute of the State of São Paulo (ICESP), Hospital das Clinicas HCFMUSP, Departmente of Gastroenterology, University of São Paulo Medical School – 17th Floor Room

Avenida Dr. Arnaldo nº 251, Cerqueira César, São Paulo-SP, CEP 01246-000

Telephone: 3893200 extension 3994

7. References

1. Woods SDS, Miitchell GJ. Delayed return of gastric emptying

after gastroenterostomy. Br J Surg 1989;76:145–148. doi: 10.1002/bjs.1800760213.

1. Doberneck RC, Berndt GA. Delayed gastric emptying after

palliative gastrojejunostomy for cancer of the pancreas. Arch

surg. 1987;122:827–829

1. Adler DG, Baron TH. Endoscopic palliation of malignant gastric outlet obstruction using self-expanding metal stents: experience in 36 patients. Am J Gastroenterol 2002;97:72-78
2. Devine HB. Basic principle and supreme difficulties in gastric surgery. Surg Gynecol Obstet 1925;40:1–16
3. Maingot R. The surgical treatment of irremovable cancer of the

pyloric segment of the stomach. Ann Surg 1936;104:161–6.

1. [Oida T](http://www.ncbi.nlm.nih.gov/pubmed?term=Oida%20T%5BAuthor%5D&cauthor=true&cauthor_uid=19333659), [Mimatsu K](http://www.ncbi.nlm.nih.gov/pubmed?term=Mimatsu%20K%5BAuthor%5D&cauthor=true&cauthor_uid=19333659), [Kawasaki A](http://www.ncbi.nlm.nih.gov/pubmed?term=Kawasaki%20A%5BAuthor%5D&cauthor=true&cauthor_uid=19333659), [Kano H](http://www.ncbi.nlm.nih.gov/pubmed?term=Kano%20H%5BAuthor%5D&cauthor=true&cauthor_uid=19333659), [Kuboi Y](http://www.ncbi.nlm.nih.gov/pubmed?term=Kuboi%20Y%5BAuthor%5D&cauthor=true&cauthor_uid=19333659), [Amano S](http://www.ncbi.nlm.nih.gov/pubmed?term=Amano%20S%5BAuthor%5D&cauthor=true&cauthor_uid=19333659). Modified Devine exclusion with vertical stomach reconstruction for gastric outlet obstruction: a novel technique. [J Gastrointest Surg.](http://www.ncbi.nlm.nih.gov/pubmed/19333659) 2009 Jul;13(7):1226-32. Epub 2009 Mar 31
2. [Kubota K](http://www.ncbi.nlm.nih.gov/pubmed?term=Kubota%20K%5BAuthor%5D&cauthor=true&cauthor_uid=17638796), [Kuroda J](http://www.ncbi.nlm.nih.gov/pubmed?term=Kuroda%20J%5BAuthor%5D&cauthor=true&cauthor_uid=17638796), [Origuchi N](http://www.ncbi.nlm.nih.gov/pubmed?term=Origuchi%20N%5BAuthor%5D&cauthor=true&cauthor_uid=17638796), [Kaminishi M](http://www.ncbi.nlm.nih.gov/pubmed?term=Kaminishi%20M%5BAuthor%5D&cauthor=true&cauthor_uid=17638796), [Isayama H](http://www.ncbi.nlm.nih.gov/pubmed?term=Isayama%20H%5BAuthor%5D&cauthor=true&cauthor_uid=17638796), [Kawabe T](http://www.ncbi.nlm.nih.gov/pubmed?term=Kawabe%20T%5BAuthor%5D&cauthor=true&cauthor_uid=17638796), [Omata M](http://www.ncbi.nlm.nih.gov/pubmed?term=Omata%20M%5BAuthor%5D&cauthor=true&cauthor_uid=17638796), [Mafune K](http://www.ncbi.nlm.nih.gov/pubmed?term=Mafune%20K%5BAuthor%5D&cauthor=true&cauthor_uid=17638796). Stomach-partitioning gastrojejunostomy for gastroduodenal outlet obstruction. [Arch Surg.](http://www.ncbi.nlm.nih.gov/pubmed/17638796) 2007 Jul;142(7):607-11
3. [Kwon SJ](http://www.ncbi.nlm.nih.gov/pubmed?term=Kwon%20SJ%5BAuthor%5D&cauthor=true&cauthor_uid=14994143), [Lee HG](http://www.ncbi.nlm.nih.gov/pubmed?term=Lee%20HG%5BAuthor%5D&cauthor=true&cauthor_uid=14994143). Gastric partitioning gastrojejunostomy in unresectable distal gastric cancer patients. [World J Surg.](http://www.ncbi.nlm.nih.gov/pubmed/14994143) 2004 Apr;28(4):365-8. Epub 2004 Mar 4.
4. [Usuba T](http://www.ncbi.nlm.nih.gov/pubmed?term=Usuba%20T%5BAuthor%5D&cauthor=true&cauthor_uid=21191698), [Misawa T](http://www.ncbi.nlm.nih.gov/pubmed?term=Misawa%20T%5BAuthor%5D&cauthor=true&cauthor_uid=21191698), [Toyama Y](http://www.ncbi.nlm.nih.gov/pubmed?term=Toyama%20Y%5BAuthor%5D&cauthor=true&cauthor_uid=21191698), [Ishida Y](http://www.ncbi.nlm.nih.gov/pubmed?term=Ishida%20Y%5BAuthor%5D&cauthor=true&cauthor_uid=21191698), [Ishii Y](http://www.ncbi.nlm.nih.gov/pubmed?term=Ishii%20Y%5BAuthor%5D&cauthor=true&cauthor_uid=21191698), [Yanagisawa S](http://www.ncbi.nlm.nih.gov/pubmed?term=Yanagisawa%20S%5BAuthor%5D&cauthor=true&cauthor_uid=21191698), [Kobayashi S](http://www.ncbi.nlm.nih.gov/pubmed?term=Kobayashi%20S%5BAuthor%5D&cauthor=true&cauthor_uid=21191698), [Yanaga K](http://www.ncbi.nlm.nih.gov/pubmed?term=Yanaga%20K%5BAuthor%5D&cauthor=true&cauthor_uid=21191698). Is modified Devine exclusion necessary for gastrojejunostomy in patients with unresectable pancreatobiliary cancer? [Surg Today.](http://www.ncbi.nlm.nih.gov/pubmed/21191698) 2011 Jan;41(1):97-100. Epub 2010 Dec 30.
5. [Ammori BJ](http://www.ncbi.nlm.nih.gov/pubmed?term=Ammori%20BJ%5BAuthor%5D&cauthor=true&cauthor_uid=12409703), [Boreham B](http://www.ncbi.nlm.nih.gov/pubmed?term=Boreham%20B%5BAuthor%5D&cauthor=true&cauthor_uid=12409703). Laparoscopic devine exclusion gastroenterostomy for the palliation of unresectable and obstructing gastric carcinoma. [Surg Laparosc Endosc Percutan Tech.](http://www.ncbi.nlm.nih.gov/pubmed/12409703) 2002 Oct;12(5):353-5
6. [Navarra G](http://www.ncbi.nlm.nih.gov/pubmed?term=Navarra%20G%5BAuthor%5D&cauthor=true&cauthor_uid=17063298), [Musolino C](http://www.ncbi.nlm.nih.gov/pubmed?term=Musolino%20C%5BAuthor%5D&cauthor=true&cauthor_uid=17063298), [Venneri A](http://www.ncbi.nlm.nih.gov/pubmed?term=Venneri%20A%5BAuthor%5D&cauthor=true&cauthor_uid=17063298), [De Marco ML](http://www.ncbi.nlm.nih.gov/pubmed?term=De%20Marco%20ML%5BAuthor%5D&cauthor=true&cauthor_uid=17063298), [Bartolotta M](http://www.ncbi.nlm.nih.gov/pubmed?term=Bartolotta%20M%5BAuthor%5D&cauthor=true&cauthor_uid=17063298) Palliative antecolic isoperistaltic gastrojejunostomy: a randomized controlled trial comparing open and laparoscopic approaches. [Surg Endosc.](http://www.ncbi.nlm.nih.gov/pubmed/17063298) 2006 Dec;20(12):1831-4.
7. [Oida T](http://www.ncbi.nlm.nih.gov/pubmed?term=Oida%20T%5BAuthor%5D&cauthor=true&cauthor_uid=19453075), [Mimatsu K](http://www.ncbi.nlm.nih.gov/pubmed?term=Mimatsu%20K%5BAuthor%5D&cauthor=true&cauthor_uid=19453075), [Kawasaki A](http://www.ncbi.nlm.nih.gov/pubmed?term=Kawasaki%20A%5BAuthor%5D&cauthor=true&cauthor_uid=19453075), [Kano H](http://www.ncbi.nlm.nih.gov/pubmed?term=Kano%20H%5BAuthor%5D&cauthor=true&cauthor_uid=19453075), [Kuboi Y](http://www.ncbi.nlm.nih.gov/pubmed?term=Kuboi%20Y%5BAuthor%5D&cauthor=true&cauthor_uid=19453075), [Amano S](http://www.ncbi.nlm.nih.gov/pubmed?term=Amano%20S%5BAuthor%5D&cauthor=true&cauthor_uid=19453075).

A novel technique of laparoscopic gastrojejunostomy-modified Devine exclusion with verticalstomach reconstruction-for gastric outlet obstruction to preventing blow-out of the distalgastric remnant and delayed in return of gastric emptying. [Hepatogastroenterology.](http://www.ncbi.nlm.nih.gov/pubmed/19453075) 2009 Jan-Feb;56(89):282-4.

1. [Chiu CC](http://www.ncbi.nlm.nih.gov/pubmed?term=Chiu%20CC%5BAuthor%5D&cauthor=true&cauthor_uid=16646703), [Wang W](http://www.ncbi.nlm.nih.gov/pubmed?term=Wang%20W%5BAuthor%5D&cauthor=true&cauthor_uid=16646703), [Huang MT](http://www.ncbi.nlm.nih.gov/pubmed?term=Huang%20MT%5BAuthor%5D&cauthor=true&cauthor_uid=16646703), [Wei PL](http://www.ncbi.nlm.nih.gov/pubmed?term=Wei%20PL%5BAuthor%5D&cauthor=true&cauthor_uid=16646703), [Chen TC](http://www.ncbi.nlm.nih.gov/pubmed?term=Chen%20TC%5BAuthor%5D&cauthor=true&cauthor_uid=16646703), [Lee WJ](http://www.ncbi.nlm.nih.gov/pubmed?term=Lee%20WJ%5BAuthor%5D&cauthor=true&cauthor_uid=16646703). Palliative gastrojejunostomy for advanced gastric antral cancer: double scope technique. [J Laparoendosc Adv Surg Tech A.](http://www.ncbi.nlm.nih.gov/pubmed/16646703) 2006 Apr;16(2):133-6.
2. [Matsumoto T](http://www.ncbi.nlm.nih.gov/pubmed?term=Matsumoto%20T%5BAuthor%5D&cauthor=true&cauthor_uid=21188010), [Izumi K](http://www.ncbi.nlm.nih.gov/pubmed?term=Izumi%20K%5BAuthor%5D&cauthor=true&cauthor_uid=21188010), [Shiromizu A](http://www.ncbi.nlm.nih.gov/pubmed?term=Shiromizu%20A%5BAuthor%5D&cauthor=true&cauthor_uid=21188010), [Shibata K](http://www.ncbi.nlm.nih.gov/pubmed?term=Shibata%20K%5BAuthor%5D&cauthor=true&cauthor_uid=21188010), [Ohta M](http://www.ncbi.nlm.nih.gov/pubmed?term=Ohta%20M%5BAuthor%5D&cauthor=true&cauthor_uid=21188010), [Kitano S](http://www.ncbi.nlm.nih.gov/pubmed?term=Kitano%20S%5BAuthor%5D&cauthor=true&cauthor_uid=21188010). Laparoscopic gastric partitioning gastrojejunostomy for an unresectable duodenal malignant tumor. [J Minim Access Surg.](http://www.ncbi.nlm.nih.gov/pubmed/21188010) 2005 Sep;1(3):129-32
3. [Mimatsu K](http://www.ncbi.nlm.nih.gov/pubmed?term=Mimatsu%20K%5BAuthor%5D&cauthor=true&cauthor_uid=19542834), [Oida T](http://www.ncbi.nlm.nih.gov/pubmed?term=Oida%20T%5BAuthor%5D&cauthor=true&cauthor_uid=19542834), [Kawasaki A](http://www.ncbi.nlm.nih.gov/pubmed?term=Kawasaki%20A%5BAuthor%5D&cauthor=true&cauthor_uid=19542834), [Kano H](http://www.ncbi.nlm.nih.gov/pubmed?term=Kano%20H%5BAuthor%5D&cauthor=true&cauthor_uid=19542834), [Kuboi Y](http://www.ncbi.nlm.nih.gov/pubmed?term=Kuboi%20Y%5BAuthor%5D&cauthor=true&cauthor_uid=19542834), [Aramaki O](http://www.ncbi.nlm.nih.gov/pubmed?term=Aramaki%20O%5BAuthor%5D&cauthor=true&cauthor_uid=19542834), [Amano S](http://www.ncbi.nlm.nih.gov/pubmed?term=Amano%20S%5BAuthor%5D&cauthor=true&cauthor_uid=19542834).Laparoscopic-assisted stomach-partitioning gastrojejunostomy for the palliation of gastricoutlet obstruction from antral gastric cancer. [Surg Laparosc Endosc Percutan Tech.](http://www.ncbi.nlm.nih.gov/pubmed/19542834) 2009 Jun;19(3):e76-9.
4. [Ohashi M](http://www.ncbi.nlm.nih.gov/pubmed?term=Ohashi%20M%5BAuthor%5D&cauthor=true&cauthor_uid=19039635), [Kanda T](http://www.ncbi.nlm.nih.gov/pubmed?term=Kanda%20T%5BAuthor%5D&cauthor=true&cauthor_uid=19039635), [Hirota M](http://www.ncbi.nlm.nih.gov/pubmed?term=Hirota%20M%5BAuthor%5D&cauthor=true&cauthor_uid=19039635), [Kobayashi T](http://www.ncbi.nlm.nih.gov/pubmed?term=Kobayashi%20T%5BAuthor%5D&cauthor=true&cauthor_uid=19039635), [Yajima K](http://www.ncbi.nlm.nih.gov/pubmed?term=Yajima%20K%5BAuthor%5D&cauthor=true&cauthor_uid=19039635), [Kosugi S](http://www.ncbi.nlm.nih.gov/pubmed?term=Kosugi%20S%5BAuthor%5D&cauthor=true&cauthor_uid=19039635), [Hatakeyama](http://www.ncbi.nlm.nih.gov/pubmed?term=Hatakeyama%20K%5BAuthor%5D&cauthor=true&cauthor_uid=19039635) K. Gastrojejunostomy as induction treatment for S-1-based chemotherapy in patients with incurable gastric cancer. [Surg Today.](http://www.ncbi.nlm.nih.gov/pubmed/19039635) 2008;38(12):1102-7. Epub 2008 Nov 28.
5. [Stupart DA](http://www.ncbi.nlm.nih.gov/pubmed?term=Stupart%20DA%5BAuthor%5D&cauthor=true&cauthor_uid=16878509), [Panieri E](http://www.ncbi.nlm.nih.gov/pubmed?term=Panieri%20E%5BAuthor%5D&cauthor=true&cauthor_uid=16878509), [Dent DM](http://www.ncbi.nlm.nih.gov/pubmed?term=Dent%20DM%5BAuthor%5D&cauthor=true&cauthor_uid=16878509). Gastrojejunostomy for gastric outlet obstruction in patients with gastric carcinoma. [S Afr J Surg.](http://www.ncbi.nlm.nih.gov/pubmed/16878509) 2006 May;44(2):52-4.
6. [Suzuki O](http://www.ncbi.nlm.nih.gov/pubmed?term=Suzuki%20O%5BAuthor%5D&cauthor=true&cauthor_uid=17693295), [Shichinohe T](http://www.ncbi.nlm.nih.gov/pubmed?term=Shichinohe%20T%5BAuthor%5D&cauthor=true&cauthor_uid=17693295), [Yano T](http://www.ncbi.nlm.nih.gov/pubmed?term=Yano%20T%5BAuthor%5D&cauthor=true&cauthor_uid=17693295), [Okamura K](http://www.ncbi.nlm.nih.gov/pubmed?term=Okamura%20K%5BAuthor%5D&cauthor=true&cauthor_uid=17693295), [Hazama K](http://www.ncbi.nlm.nih.gov/pubmed?term=Hazama%20K%5BAuthor%5D&cauthor=true&cauthor_uid=17693295), [Hirano S](http://www.ncbi.nlm.nih.gov/pubmed?term=Hirano%20S%5BAuthor%5D&cauthor=true&cauthor_uid=17693295), [Kondo S](http://www.ncbi.nlm.nih.gov/pubmed?term=Kondo%20S%5BAuthor%5D&cauthor=true&cauthor_uid=17693295). Laparoscopic modified Devine exclusion gastrojejunostomy as a palliative surgery to relieve malignant pyloroduodenal obstruction by unresectable cancer. [Am J Surg.](http://www.ncbi.nlm.nih.gov/pubmed/17693295) 2007 Sep;194(3):416-8.
7. Kum CK, Yap CH, Goh PM. Palliation of advanced gastric câncer by laparoscopic gastrojejunostomy. Singapore Med J 1995;36: 228-9
8. [Yamagishi F](http://www.ncbi.nlm.nih.gov/pubmed?term=Yamagishi%20F%5BAuthor%5D&cauthor=true&cauthor_uid=15532791), [Arai H](http://www.ncbi.nlm.nih.gov/pubmed?term=Arai%20H%5BAuthor%5D&cauthor=true&cauthor_uid=15532791), [Yoshida T](http://www.ncbi.nlm.nih.gov/pubmed?term=Yoshida%20T%5BAuthor%5D&cauthor=true&cauthor_uid=15532791), [Tyou S](http://www.ncbi.nlm.nih.gov/pubmed?term=Tyou%20S%5BAuthor%5D&cauthor=true&cauthor_uid=15532791), [Nagata T](http://www.ncbi.nlm.nih.gov/pubmed?term=Nagata%20T%5BAuthor%5D&cauthor=true&cauthor_uid=15532791), [Bando T](http://www.ncbi.nlm.nih.gov/pubmed?term=Bando%20T%5BAuthor%5D&cauthor=true&cauthor_uid=15532791), [Abe H](http://www.ncbi.nlm.nih.gov/pubmed?term=Abe%20H%5BAuthor%5D&cauthor=true&cauthor_uid=15532791), [Tsukada K](http://www.ncbi.nlm.nih.gov/pubmed?term=Tsukada%20K%5BAuthor%5D&cauthor=true&cauthor_uid=15532791). Partial separating gastrojejunostomy for incurable cancer of the stomach or pancreas. [Hepatogastroenterology.](http://www.ncbi.nlm.nih.gov/pubmed/15532791) 2004 Nov-Dec;51(60):1623-5.
9. [Arciero CA](http://www.ncbi.nlm.nih.gov/pubmed?term=Arciero%20CA%5BAuthor%5D&cauthor=true&cauthor_uid=16490561), [Joseph N](http://www.ncbi.nlm.nih.gov/pubmed?term=Joseph%20N%5BAuthor%5D&cauthor=true&cauthor_uid=16490561), [Watson JC](http://www.ncbi.nlm.nih.gov/pubmed?term=Watson%20JC%5BAuthor%5D&cauthor=true&cauthor_uid=16490561), [Hoffman JP](http://www.ncbi.nlm.nih.gov/pubmed?term=Hoffman%20JP%5BAuthor%5D&cauthor=true&cauthor_uid=16490561) Partial stomach-partitioning gastrojejunostomy for malignant duodenal obstruction. [Am J Surg.](http://www.ncbi.nlm.nih.gov/pubmed/16490561) 2006 Mar;191(3):428-32.
10. [Farooq A](http://www.ncbi.nlm.nih.gov/pubmed?term=Farooq%20A%5BAuthor%5D&cauthor=true&cauthor_uid=15532850), [Patel R](http://www.ncbi.nlm.nih.gov/pubmed?term=Patel%20R%5BAuthor%5D&cauthor=true&cauthor_uid=15532850), [Sorefan N](http://www.ncbi.nlm.nih.gov/pubmed?term=Sorefan%20N%5BAuthor%5D&cauthor=true&cauthor_uid=15532850), [Ammori BJ](http://www.ncbi.nlm.nih.gov/pubmed?term=Ammori%20BJ%5BAuthor%5D&cauthor=true&cauthor_uid=15532850). Laparoscopic exclusion gastroenterostomy for palliation of gastric outlet obstruction secondary to recurrent cholangiocarcinoma. [Hepatogastroenterology.](http://www.ncbi.nlm.nih.gov/pubmed/15532850) 2004 Nov-Dec;51(60):1886-8.
11. [Jeurnink SM](http://www.ncbi.nlm.nih.gov/pubmed?term=Jeurnink%20SM%5BAuthor%5D&cauthor=true&cauthor_uid=20003966), [Steyerberg EW](http://www.ncbi.nlm.nih.gov/pubmed?term=Steyerberg%20EW%5BAuthor%5D&cauthor=true&cauthor_uid=20003966), [van Hooft JE](http://www.ncbi.nlm.nih.gov/pubmed?term=van%20Hooft%20JE%5BAuthor%5D&cauthor=true&cauthor_uid=20003966), [van Eijck CH](http://www.ncbi.nlm.nih.gov/pubmed?term=van%20Eijck%20CH%5BAuthor%5D&cauthor=true&cauthor_uid=20003966), [Schwartz MP](http://www.ncbi.nlm.nih.gov/pubmed?term=Schwartz%20MP%5BAuthor%5D&cauthor=true&cauthor_uid=20003966), [Vleggaar FP](http://www.ncbi.nlm.nih.gov/pubmed?term=Vleggaar%20FP%5BAuthor%5D&cauthor=true&cauthor_uid=20003966), [Kuipers EJ](http://www.ncbi.nlm.nih.gov/pubmed?term=Kuipers%20EJ%5BAuthor%5D&cauthor=true&cauthor_uid=20003966), [Siersema PD](http://www.ncbi.nlm.nih.gov/pubmed?term=Siersema%20PD%5BAuthor%5D&cauthor=true&cauthor_uid=20003966); [Dutch SUSTENT Study Group](http://www.ncbi.nlm.nih.gov/pubmed?term=Dutch%20SUSTENT%20Study%20Group%5BCorporate%20Author%5D). Surgical gastrojejunostomy or endoscopic stent placement for the palliation of malignant gastric outlet obstruction (SUSTENT study): a multicenter randomized trial. [Gastrointest Endosc.](http://www.ncbi.nlm.nih.gov/pubmed/20003966) 2010 Mar;71(3):490-9. Epub 2009 Dec 8.
12. [Moura EG](http://www.ncbi.nlm.nih.gov/pubmed?term=Moura%20EG%5BAuthor%5D&cauthor=true&cauthor_uid=22408353), [Ferreira FC](http://www.ncbi.nlm.nih.gov/pubmed?term=Ferreira%20FC%5BAuthor%5D&cauthor=true&cauthor_uid=22408353), [Cheng S](http://www.ncbi.nlm.nih.gov/pubmed?term=Cheng%20S%5BAuthor%5D&cauthor=true&cauthor_uid=22408353), [Moura DT](http://www.ncbi.nlm.nih.gov/pubmed?term=Moura%20DT%5BAuthor%5D&cauthor=true&cauthor_uid=22408353), [Sakai P](http://www.ncbi.nlm.nih.gov/pubmed?term=Sakai%20P%5BAuthor%5D&cauthor=true&cauthor_uid=22408353), [Zilberstein B](http://www.ncbi.nlm.nih.gov/pubmed?term=Zilberstain%20B%5BAuthor%5D&cauthor=true&cauthor_uid=22408353). Duodenal stenting for malignant gastric outlet obstruction: prospective study. [World J Gastroenterol.](http://www.ncbi.nlm.nih.gov/pubmed/22408353) 2012 Mar 7;18(9):938-43.
13. Wente MN, Bassi C, Dervenis C, Fingerhut A, Gouma DJ, Izbicki JR, et al. Delayed gastric emptying (DGE) after pancreatic surgery: a suggested definition by the International Study Group of Pancreatic Surgery (ISGPS). Surgery 2007;142:761-8.
14. Dindo D, Demartines N, Clavien PA (2004) Classification of surgical complications: a new proposal with evaluation in a cohort of 6336 patients and results of a survey. Ann Surg 240:205–213
15. Japanese Gastric Cancer Association. Japanese classification of gastric carcinoma: 3rd English edition. Gastric Cancer 2011. doi:10.1007/s10120-011-0041-5.
16. Japanese Gastric Cancer Association. Japanese Gastric Cancer Treatment Guidelines 2010 (ver. 3). Gastric Cancer 2011. doi: 10.1007/s10120-011-0042-4.

8. Informed consent form (in Portuguese language)


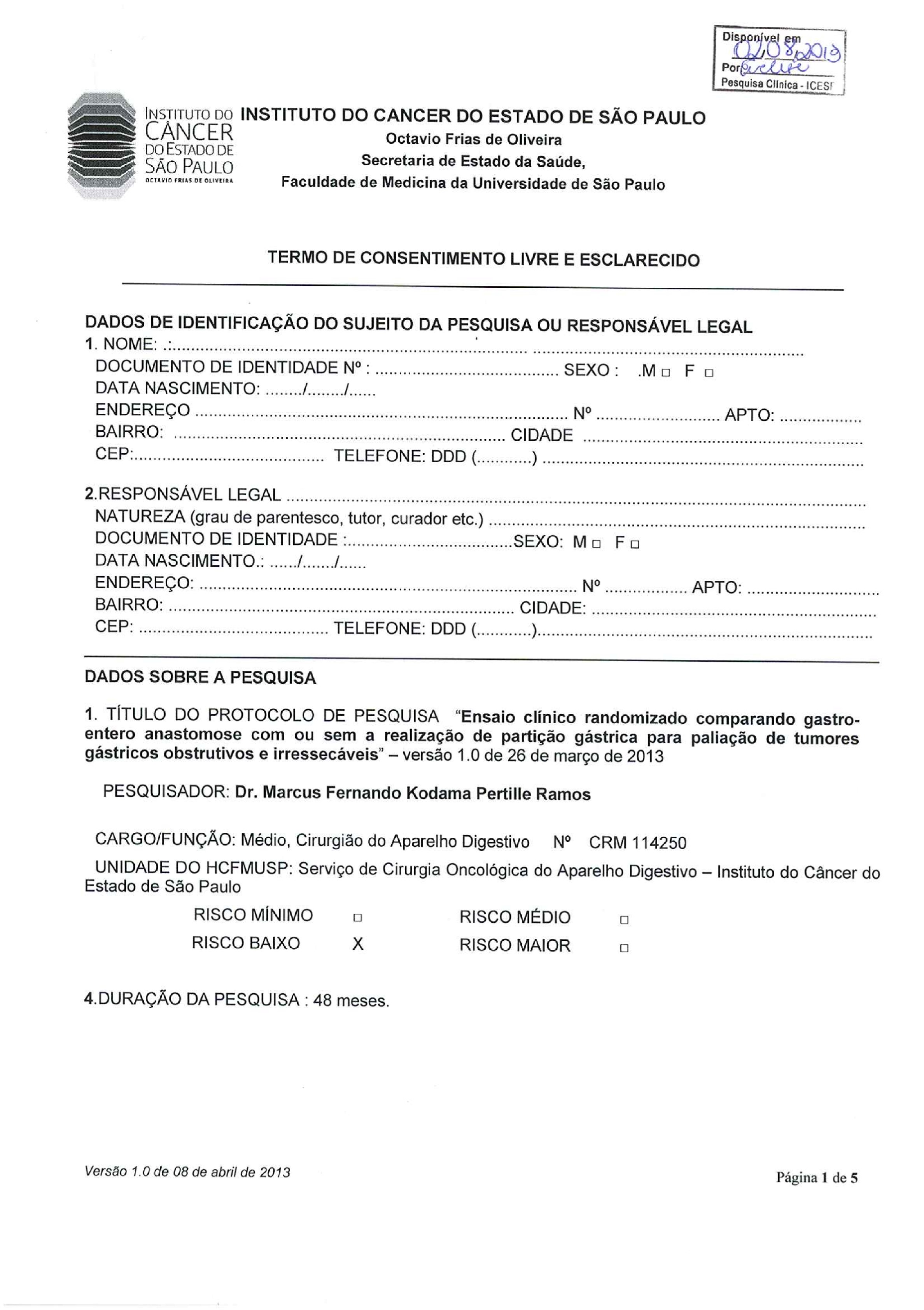
8. Informed consent form (in Portuguese language)


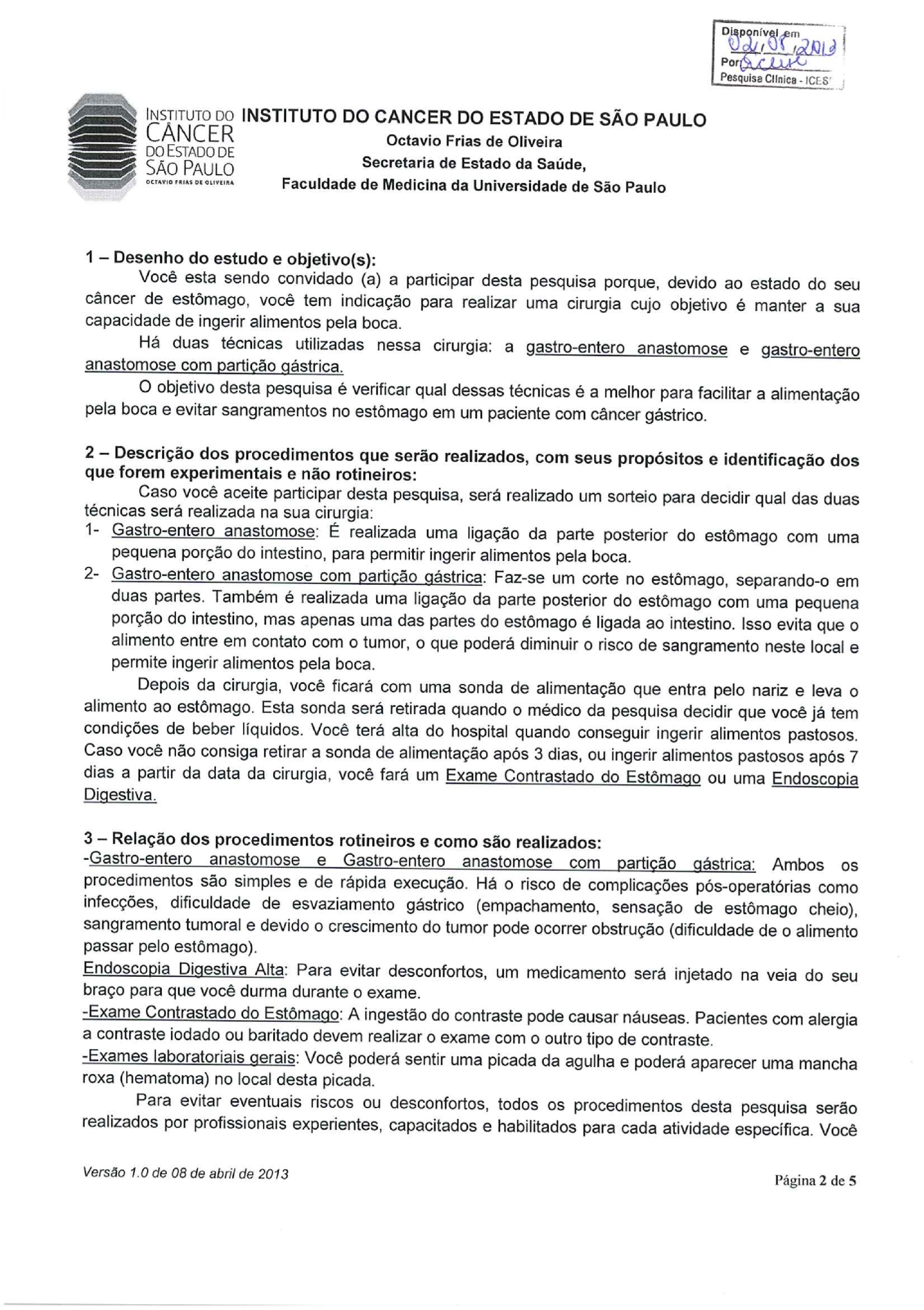


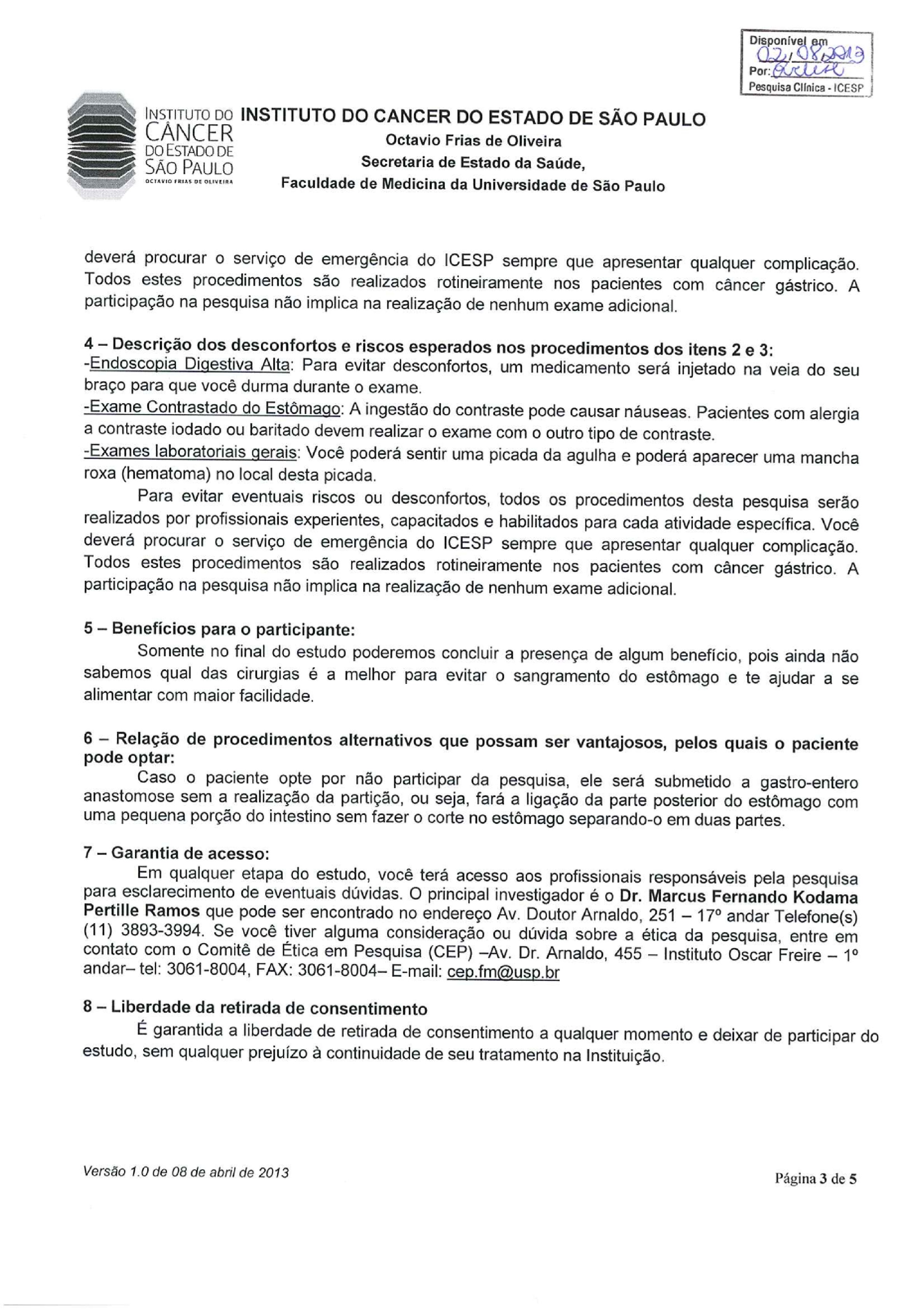


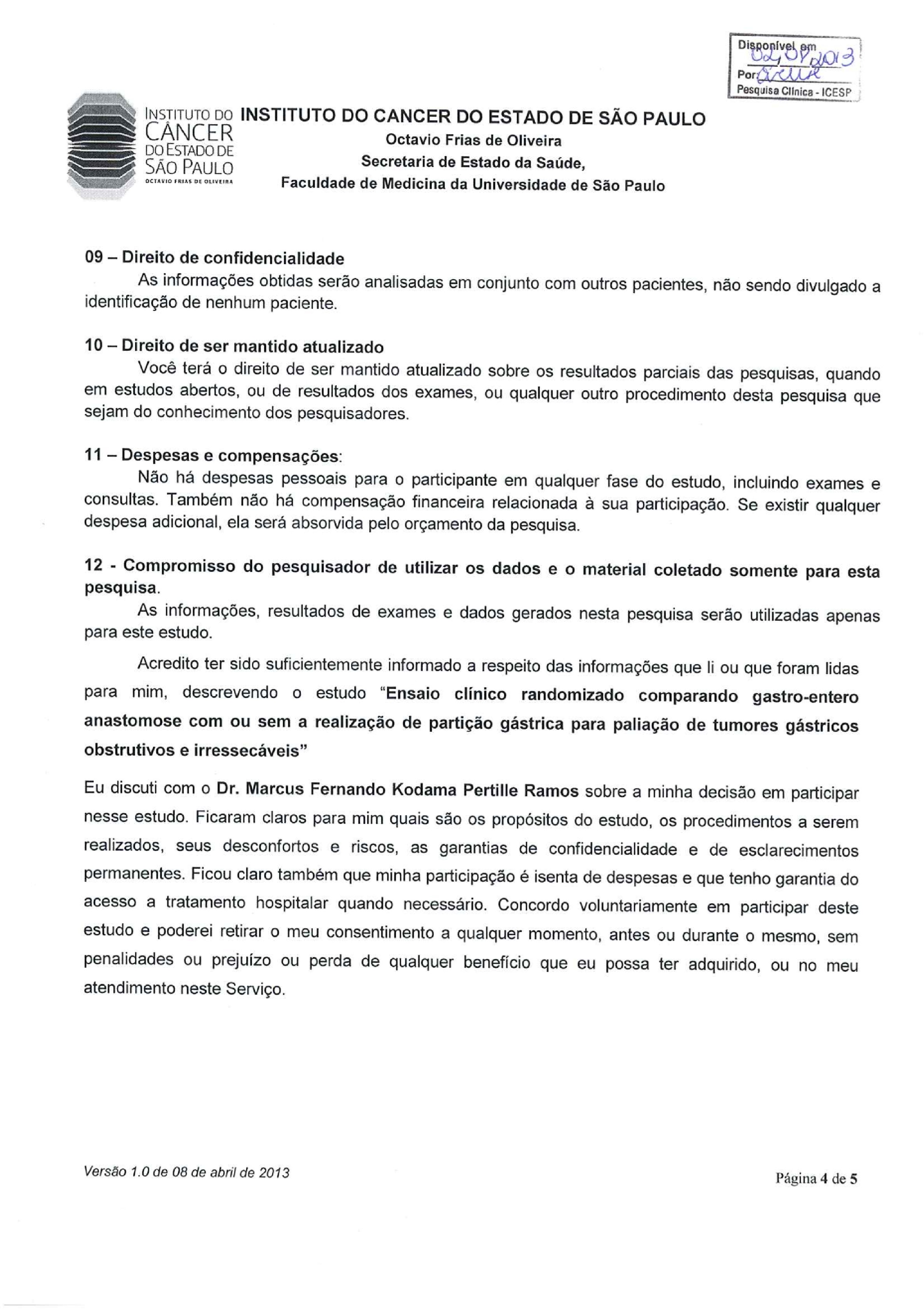


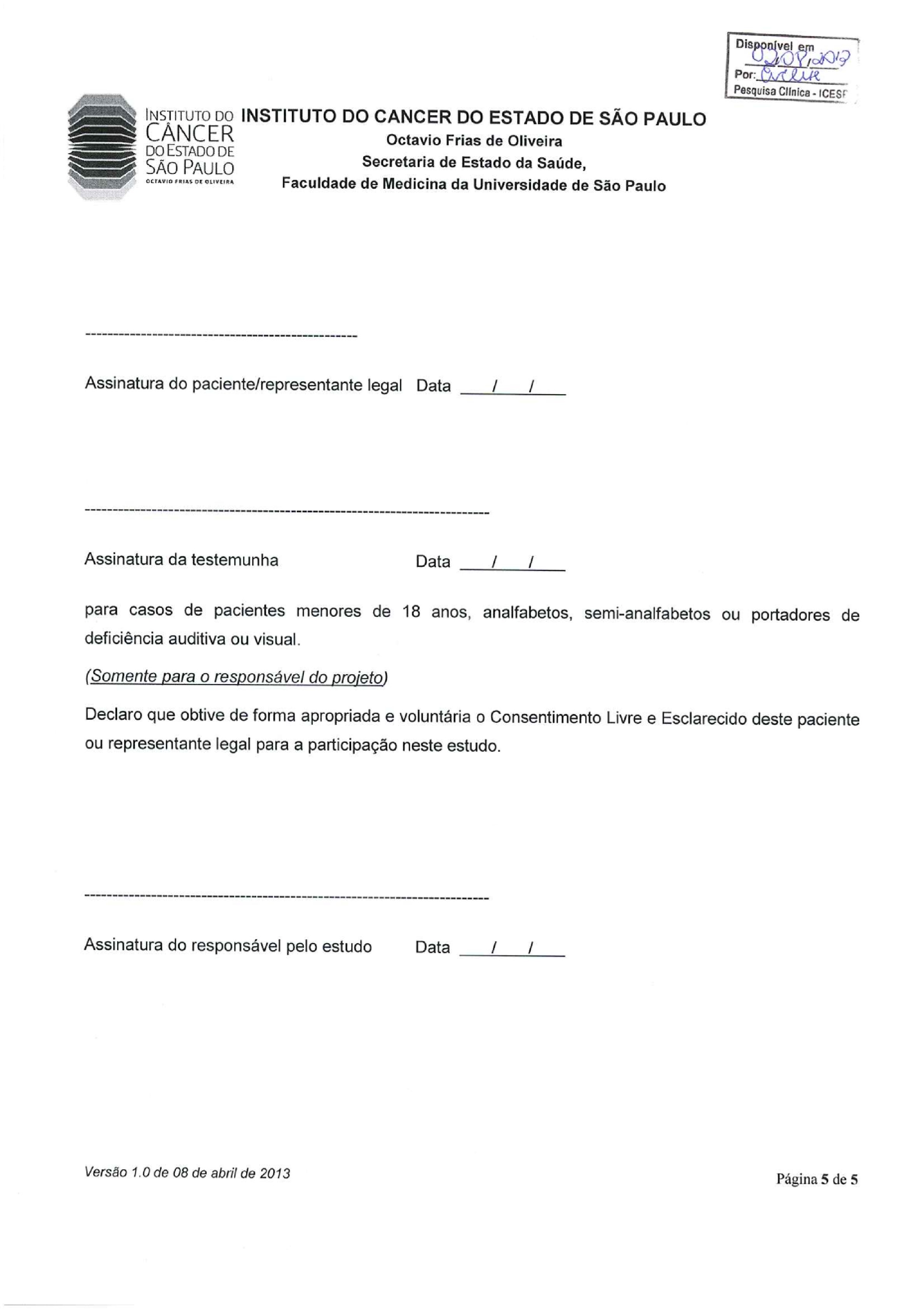

Supplement: zrae152_Supplementary_Data [file zrae152_supplementary_data.zip › Original study protocol 2014 for BJS 2024.docx]
